# Deciphering the interplay between inflammation and dysregulated autophagy in lupus nephritis through network analysis and experimental validation

**DOI:** 10.3389/fcell.2026.1764975

**Published:** 2026-01-14

**Authors:** Runrun Zhang, Xin Lv, Qice Sun, Wenhan Huang, Ting Zhao

**Affiliations:** 1 The Second Affiliated Hospital of Zhejiang Chinese Medical University, The Second Clinical Medical College, Zhejiang Chinese Medical University, Hangzhou, Zhejiang, China; 2 Department of Rheumatology, The Second Affiliated Hospital of Zhejiang Chinese Medical University, Hangzhou, China

**Keywords:** autophagy, EGFR, lupus nephritis, network analysis, experimental validation

## Abstract

**Background:**

Autophagy dysregulation plays an important role in the development and progression of lupus nephritis (LN). However, the key autophagy-related genes involved in LN and their underlying cellular mechanisms remain unclear. This study aims to systematically explore the autophagy-related molecular signatures of LN and to elucidate the relevant mechanisms.

**Methods:**

Transcriptomic data from LN and control kidney tissues were analyzed to identify differentially expressed genes (DEGs), followed by KEGG, GSEA, and GSVA enrichment. Autophagy-related DEGs (ARDEGs) were obtained by intersecting DEGs with autophagy gene sets. Hub genes were screened using PPI network analysis, cytoHubba algorithms, and WGCNA. Diagnostic performance was assessed by ROC curves and a nomogram. Single-cell datasets and qRT-PCR, pathology, TEM, and immunohistochemistry were used for validation. Functional assays were conducted in CIHP-1 podocytes with stable EGFR overexpression.

**Results:**

A total of 445 ARDEGs were identified, enriched in autophagy, PI3K-Akt/mTOR, and MAPK pathways. Eleven hub genes were obtained, among which EGFR and RAF1 showed strong diagnostic value (AUC >0.90) and correlations with immune infiltration. Single-cell and experimental validation revealed elevated EGFR expression in LN. EGFR-overexpressing podocytes exhibited increased MDC fluorescence by flow cytometry, autophagosome accumulation by TEM, and a significant increase in LC3-positive puncta by confocal microscopy.

**Conclusion:**

EGFR is a key regulatory factor related to autophagy in LN. The excessive activation of EGFR affects the autophagy of LN podocytes, providing new mechanistic insights and potential therapeutic targets for LN.

## Introduction

Lupus nephritis (LN) is one of the most severe complications of systemic lupus erythematosus (SLE), causing substantial morbidity and mortality (Tsokos, 2011; Chen et al., 2022). Although current immunosuppressive regimens have improved short-term outcomes, a substantial proportion of patients progress to renal failure, highlighting critical gaps in our understanding of LN pathogenesis. Identifying key molecular pathways that drive renal inflammation and tissue injury is therefore essential for the development of more effective diagnostic biomarkers and targeted therapies.

Autophagy refers to a cellular mechanism for breaking down and recycling proteins and organelles, thereby helping to maintain homeostasis within the cell. While typically considered a protective process, autophagy can contribute to cell death when its regulatory mechanisms are disrupted or when autophagic activity becomes excessive. (Glick et al., 2010; Klionsky et al., 2021). Accumulating evidence indicates that autophagy plays an important role in immune regulation and renal physiology, and its dysregulation has been implicated in the pathogenesis of SLE and LN (Podestà et al., 2022). Experimental studies have demonstrated that altered autophagic activity influences lymphocyte activation (Arbogast and Gros, 2018), inflammatory signaling (Monkkonen and Debnath, 2018; Deretic, 2021), and podocyte survival (Wang et al., 2024; Zhong et al., 2024), all of which are central to LN development. In addition, genetic and pharmacological modulation of autophagy-related pathways has been shown to ameliorate lupus-like phenotypes in animal models (Yu et al., 2019; Lv et al., 2022; Zhao et al., 2023), suggesting that autophagy may represent a promising therapeutic target.

Computational approaches integrating large-scale omics data provide unbiased strategies to unravel disease biology on a system-wide level and nominate candidate genes for further study (Akalin, 2006). This pioneering systems biology strategy is powerful yet remarkably underutilized in the study of LN. Through comprehensive bioinformatics investigation, we can gain completely new insights into the genetic basis of aberrant autophagy activation in LN.

In the present study, we performed an integrative bioinformatics analysis of renal transcriptomic data to systematically characterize autophagy-related gene expression patterns and regulatory networks in LN. We hypothesized that LN is associated with distinct and coordinated alterations in autophagy-related genes that contribute to renal inflammation and tissue injury. By combining advanced computational analyses with experimental validation, this work aims to identify key autophagy-associated genes and pathways that have been overlooked by previous studies, thereby providing new insights into LN pathogenesis and a foundation for future biomarker discovery and therapeutic development.

## Materials and methods

### Obtainment and preprocessing of LN datasets

Gene Expression Profiling data of LN patients were adopted from the GEO database (http://www.ncbi.nlm.nih.gov/geo). The GSE112943 dataset, generated by the GPL10558 platform, had 14 Lupus nephritis 14 samples and 7 kidney control samples. The GSE32591 dataset, generated on the GPL14663 platform, included 64 renal tissue samples from patients with lupus nephritis and 29 samples from healthy controls.

### Identification of differentially expressed genes (DEGs)

DEGs were performed with the “DESeq” R package (Anders and Huber, 2010). Adjust P value (False Discovery Rate, FDR) < 0.05 and |log2FoldChange| ≥1 were the thresholds for significant differential expression.

### KEGG pathway analysis of DEGs

The KEGG database provides information regarding molecular and higher-level gene functions, including biochemical pathways (Kanehisa et al., 2017). Annotation and visualization were performed using the “clusterProfiler” package (Yu et al., 2012) (an R package for comparing biological themes among gene clusters). Enrichment analysis was conducted with the hypergeometric test. FDR <0.05 was chosen as the cutoff criterion, indicating a statistically significant difference.

### Identification of autophagy-related DEGs (ARDEGs)

Autophagy-related genes (ARGs) were downloaded from the Human Autophagy Database (http://autophagy.lu/) (Di et al., 2023) and the MsigDB Database (https://www.gsea-msigdb.org/gsea/msigdb) (Yan et al., 2023). The R package “VennDiagram” plots a Venn diagram by overlapping DEGs with ARGs to obtain the autophagy-related DEGs (ARDEGs).

### Gene set enrichment analysis (GSEA) and gene set variation analysis (GSVA)

Gene set enrichment analysis (GSEA) is a threshold-free method that analyzes all genes based on their differential expression rank or other score without prior gene filtering (Reimand et al., 2019). The “clusterProfiler” R package was adopted for analysis, and the data set was analyzed from the MSigDB downloaded from the GSEA-MSigDB website (Zhang et al., 2022). The MSigDB database provides gene sets for performing gene set enrichment analysis (Liberzon et al., 2015). |Normalized Enrichment Score (NES)|>1, *P* < 0.05, false discovery rate (FDR)≤0.25 were selected as the cut-off criteria indicating statistically significant differences. C5: ontology gene sets are chosen for our study.

Gene Set Variation Analysis (GSVA) is a GSE method that estimates the variation of pathway activity over a sample population in an unsupervised manner (Hänzelmann et al., 2013). The “GSVA” R package investigated the biological process between the two groups (Huang et al., 2022). *P* < 0.05 was considered statistically significant. C2: KEGG subset.

### The protein-protein interaction network constructed, and hub genes identified

The protein-protein interaction (PPI) network of ARDEGs was constructed by the STRING database. STRING can describe user gene lists and allow for the creation and sharing of highly customized and enhanced protein-protein association networks (Szklarczyk et al., 2019; Zhang et al., 2022). The “minimum required interaction score” is high confidence (0.7). PPI networks were analyzed and visualized with Cytoscape (v3.10.1) software. The five significant modules were identified with the MCODE plugin, which is used to identify densely connected regions by clustering a given network based on its topology. The six algorithms in the cytoHubba plugin, including Closeness, Degree, EPC, MCC, MNC, and Radiality, were used to further obtain hub genes. The final hub genes were obtained by intersecting the hub genes obtained by seven different methods.

### The weighted gene coexpression network analysis and hub gene identified

Weighted gene coexpression network analysis (WGCNA) is adopted to create the gene coexpression network and identify the functional modules (Langfelder and Horvath, 2008). The minModuleSize = 25. The Pearson correlation analysis identifies the module with the strongest association with LN.

### Hub genes identified and correlation analysis of gene expression

The expression correlation of ARDEGs in all and LN samples was calculated. The correlations were performed by Spearman correlation analysis with the R “cor” function and visualized with the “corrplot” R package.

### Analysis of immune-infiltrated cells and the correlation with hub genes

CIBERSORT was shown to transform a normalized gene expression matrix into the composition of 22 immune cell types based on a deconvolution algorithm (Newman et al., 2015). The CIBERSORT algorithm was adopted to analyze the GSE112943 immune infiltrating cell profile. The algorithm employed the LM22 signature and 1,000 permutations. *P* < 0.05 was considered statistically significant.

Spearman’s rank correlation coefficient was conducted to analyze the correlations between hub genes and immune infiltrating cells. Correlation coefficients(ρ)>0.5 and *P* < 0.05 were statistically significant.

### Friends analysis

Friends analysis is a method used to compare the similarities between different genes or gene sets. It is a semantic similarity measure based on Gene Ontology, which uses the functional information of genes to analyze their correlation (Tian et al., 2023). The “GOSemSim” R package was adopted for the Friends analysis to select the most essential genes from hub genes (Yu et al., 2010).

### Regression analysis and the construction of prediction models

Univariate analysis was adopted to explore the relationship between hub genes and LN. For better performance in diagnosis and prediction, a nomogram was constructed on the hub genes (Zhu et al., 2023). The nomogram was built using the “rms” R package (Harrell, 2017). The area under the receiver operating characteristic (ROC) curve was adopted to analyze each hub gene and the nomogram in diagnosing LN (Obuchowski and Bullen, 2018). The calibration curve is used to estimate whether the predicted values of the nomogram are consistent with the actual observed values (Wu et al., 2023). The more the calibration curve coincides with the baseline, the more consistent the predicted values of the nomogram are with the existing experimental values (Tan et al., 2022).

### Single-cell RNA sequencing profiling

Single-cell RNA sequencing (scRNA-seq) can provide a deep understanding of gene-gene associations or transcriptional networks between cell populations based on the sequencing of many cells (Dai et al., 2019). ScRNA-seq enables studies of similar cell types far from the site of kidney injury to identify novel biomarkers (Rao et al., 2020). The scRNA-seq data were obtained from the ImmPort repository (accession SDY997) (Fava et al., 2011). The R package “Seurat” was employed for unsupervised clustering of single cells from LN and control samples, and the SingleR was employed for annotation (Huang et al., 2021). The UMAP clustering methods for dimensionality reduction were used in this study.

### Animals

Female MRL/lpr mice (six to eight weeks) and female C57BL/6 mice (six to eight weeks), with an average body weight of 20 ± 2 g, were obtained from Shanghai SLAC Laboratory Animal Co., Ltd. (Animal Production License No. SCXK [Shanghai] 2017–0005). All animals were maintained under specific pathogen-free (SPF) conditions at the Animal Experimental Research Center of Zhejiang Chinese Medicine University (Experimental Animal License No. SYXK [Zhejiang] 2018-0012). All experimental procedures were reviewed and approved by the Institutional Animal Care and Use Committee of Zhejiang Chinese Medicine University (Approval No. IACUC-20211,108-12). After 8 weeks of housing, mice in both groups were euthanized, and kidney tissues were harvested for subsequent analyses.

### Histopathological staining and evaluation

Renal tissues were fixed in 10% neutral buffered formalin for 24 h, followed by dehydration through a graded ethanol series (70%, 80%, 95%, and 100%), clearing in xylene, and embedding in paraffin. Sections of 4 μm thickness were cut using a rotary microtome; For Hematoxylin and Eosin (H&E) staining, the Beyotime H&E Staining Kit (Product No. C0105S) was used according to the manufacturer’s instructions. Sections were sequentially stained with Harris hematoxylin and eosin Y, dehydrated, cleared, and mounted; For Masson’s trichrome staining, the Beyotime Masson’s Trichrome Staining Kit (Product No. C0142S) was employed. Sections were treated sequentially with Weigert’s iron hematoxylin, Biebrich scarlet-acid fuchsin, phosphomolybdic/phosphotungstic acid solution, and aniline blue, followed by differentiation in acetic acid, dehydration, clearing, and mounting, as per the kit protocol; For Periodic Acid–Schiff (PAS) staining, the Beyotime PAS Staining Kit (Product No. C0189S) was utilized. Sections were oxidized in periodic acid solution, stained with Schiff reagent, counterstained with hematoxylin, and then dehydrated, cleared, and mounted following the manufacturer’s guidelines. All stained slides were examined and imaged using a light microscope.

Histopathological evaluation focused on the following characteristic lesions:

Glomerular Lesions: Endocapillary Hypercellularity: Proliferation of mesangial and endothelial cells, accompanied by polymorphonuclear neutrophil infiltration; Crescent Formation: Presence of cellular crescents within Bowman’s capsule; Hyaline Thrombi: Eosinophilic, PAS-positive occlusive material within glomerular capillary lumens (H&E, PAS); Immune Complex Deposition: Subendothelial deposits visible as eosinophilic “hyaline” material (H&E), PAS-positive material, or fuchsinophilic deposits resembling “wire loops” (Masson’s trichrome); Tubulointerstitial Damage: Tubular Atrophy and Interstitial Inflammation: Assessed based on tubular dilation, epithelial flattening, and mononuclear cell infiltration in the interstitium; Interstitial Fibrosis: Identified by areas of blue collagen deposition in Masson’s trichrome-stained sections; Vascular Lesions: Presence of segmental fibrinoid necrosis in glomerular capillaries and intravascular hyaline thrombi.

### Cell culture and processing

The immortal human derived renal podocyte cell line CIHP-1 was purchased from Beijing Fubo Biotechnology Co., Ltd. (Product No. FBCL2106). The cells were cultured in RPMI-1640 complete medium containing 10% fetal bovine serum and 1% penicillin streptomycin dual antibody, and placed in a constant temperature incubator at 37 °C and 5% CO_2_.

### Quantitative real-time polymerase chain reaction (qRT-PCR)

The qRT-PCR was adopted to validate the reliability of the hub genes. Total RNA was extracted from the kidney tissue of 6 MRL/lpr mice and 6 C57BL/6 mice with TRIzol reagent (Product No. 15596018CN, Invitrogen, Thermo Fisher Scientific, Inc.). The RNA was reverse transcribed to cDNA with HiScript® III RT SuperMix for qPCR (+gDNA wiper) (Product No. MH102, Vazyme, Nanjing, China), and qRT-PCR was carried out with ChamQ Blue Universal SYBR qPCR Master Mix (Product No. MH101, Vazyme). GAPDH was adopted as a reference. The relative mRNA expression level was calculated using the 2^-△△^Ct method, and three replicate experiments were involved. *P* < 0.05 indicated a significant difference. The primer sequences were as follows: RAF1-F-Mouse: TGG​AAT​GAG​CTT​ACA​TGA​CTG​C, RAF1-R-Mouse: GTT​GTG​AGT​GGA​ACA​TGA​TCC​AA; EGFR-F-Mouse: CAA​TGT​TCC​CAT​CGC​TGT​CGT, EGFR-R-Mouse: TGT​CTT​TGC​ATG​TGG​CCT​CAT.

### Lentiviral construction and establishment of stable cell lines

The full-length coding sequence of human EGFR (NM_005228.4) was synthesized by GeneChem Co., Ltd. And cloned into the lentiviral vector pLVX-Puro. The empty vector was used as a negative control.

CIHP-1 cells were seeded into 6-well plates at a density of 5 × 10^4 cells per well and cultured for 24 h until reaching approximately 50% confluence. Lentiviral particles were added at a multiplicity of infection (MOI = 20) along with Polybrene at a final concentration of 6 μg/mL to enhance infection efficiency. After 8 h of infection, the medium was replaced with fresh complete medium. At 48 h post-infection, the medium was replaced with complete medium containing 2 μg/mL puromycin for selection. Selection was continued for 7–10 days, until the control (non-infected) cells were eliminated. The surviving cells were considered stable EGFR-overexpressing cell lines, and EGFR expression levels were verified by qRT-PCR.

### Detection of autophagy levels by monodansylcadaverine (MDC) staining

Autophagic vacuoles were stained using an Autophagy Detection Kit (MDC method, No. C3018S, Beyotime). Control and EGFR-overexpression CIHP-1 cells were seeded into 12-well plates and cultured to a normal growth state. A total of 500 μL of MDC staining working solution was added to each well, followed by incubation in a 37 °C cell incubator for 30 min in the dark. After incubation, the MDC solution was completely removed, and the unbound dye was washed away using pre-cooled Assay Buffer. Samples were immediately analyzed using a flow cytometer, with the excitation wavelength (Ex) set to 335 nm and the emission wavelength (Em) set to 512 nm.

### Transmission electron microscopy (TEM) observation of autophagic structures

For kidney tissue, small cortical fragments (≈1 mm^3^) were immediately immersed in 2.5% glutaraldehyde at 4 °C for overnight fixation, followed by post-fixation in 1% osmium tetroxide at 4 °C for 2 h. For cultured cells, control and EGFR-overexpressing cells were gently collected using a cell scraper and centrifuged into pellets. The cell pellets were fixed in 2.5% glutaraldehyde at 4 °C for 4 h, followed by post-fixation in 1% osmium tetroxide at 4 °C for 2 h. All samples were then dehydrated through a graded ethanol series (50%, 70%, 80%, 90%, 95%, 100%), transitioned with propylene oxide, and infiltrated and embedded in Epon 812 epoxy resin. Ultrathin sections (70 nm) were cut using an ultramicrotome and double-stained with uranyl acetate and lead citrate. Samples were observed and photographed using an HT7800 transmission electron microscope (Hitachi).

### mRFP-GFP-LC3 dual-fluorescence lentiviral infection and confocal microscopy analysis

The mRFP-GFP-LC3 dual-fluorescence lentiviral reporter system (Product No. HB-LP2100001, Han Heng Bio. Shanghai, China) was used. Established control and EGFR-overexpressing CIHP-1 stable cell lines were infected again with the mRFP-GFP-LC3 lentivirus at a multiplicity of infection (MOI) = 10. After 48 h, cells were selected using dual-antibiotic medium containing 1 μg/mL puromycin and 400 μg/mL G418, resulting in stable cell lines expressing the dual-fluorescence reporter protein.

Cells were fixed with 4% paraformaldehyde, and nuclei were stained with Hoechst (Product No. C1011, Beyotime). Images were acquired using a Leica TCS SP8 laser scanning confocal microscope. GFP was excited at 488 nm and emission collected at 500–550 nm; mRFP was excited at 561 nm and emission collected at 580–630 nm.

### Statistical analysis

All data are presented as the mean ± standard deviation (SD) from a minimum of three independent experiments. Statistical comparisons were conducted using one-way ANOVA in Prism 9.0 (GraphPad, San Diego, USA). Differences with a P value <0.05 were considered statistically significant.

## Results

### DEGs, ARDEGs identified, and enrichment analysis

**FIGURE 1:**
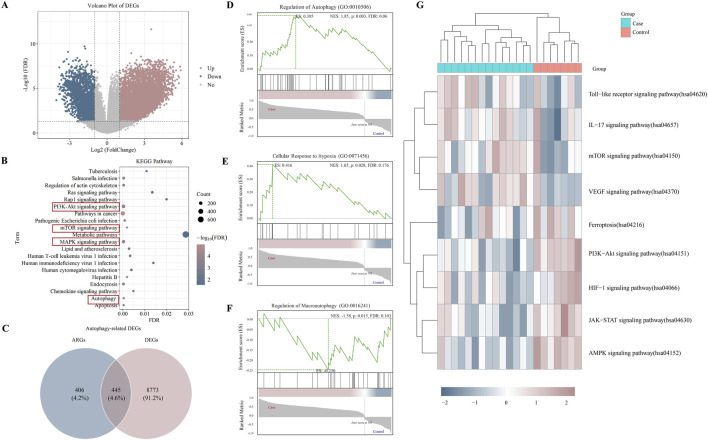
DEGs, ARDEGs identified, and enrichment analysis **(A)**, the volcano plot of DEGs. Pink: upregulated genes, Blue: downregulated genes; **(B)**, KEGG pathway enrichment terms; **(C)**, the Venn Diagram of ARDEGs; **(D–F)**, the GSEA of ARDEGs; **(G)**, the GSVA of ARDEGs.

We identified 9219 DEGs with FDR<0.05 and |log2FoldChange| ≥1. Among the DEGs, 6,875 were upregulated genes, and 2,343 were downregulated genes (Figure 1A). With KEGG analysis, the top twenty pathways include Autophagy, mTOR signaling pathway, MAPK signaling pathway, PI3K-Akt signaling pathway, and so on (Figure 1B). Subsequently, we extracted 851 ARGs from the HADb and MSigDB databases. Afterward, we obtained 445 ARDEGs through intersection analysis of the Venn diagram (Figure 1C).

To further enrich and analyze ARDEGs, GSEA results showed that ARDEGs in LN were correlated with the function of autophagy (NES = 1.85, *P* = 0.003, FDR = 0.06), hypoxia (NES = 1.65, *P* = 0.028, FDR = 0.18), and macroautophagy (NES = −1.58, *P* = 0.015, FDR = 0.10) (Figures 1D–F). GSVA results showed that ARDEGs in LN were enriched in autophagy-related pathways, such as the mTOR signaling pathway and PI3K-Akt signaling pathway (Figure 1G).

### Hub genes identified

**FIGURE 2:**
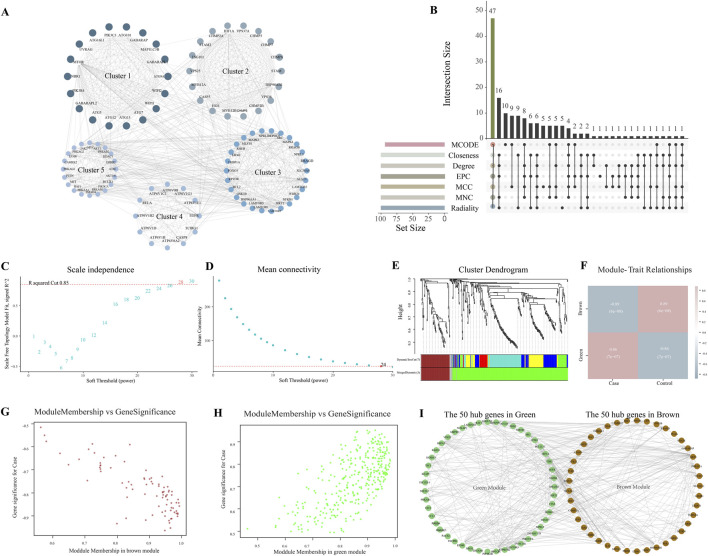
Hub genes identified **(A)**, The network constructed by MOCODE of Cytoscape; **(B)**, The hub genes identified by six algorithms of Cytoscape; **(C)**, Plot of scale independence; **(D)**, Plot of mean connectivity; **(E)**, Cluster dendrogram of the co-expression network modules; **(F)**, Module Trait Heatmap; **(G)**, Scatter plot analysis of the brown module; **(H)**, Scatter plot analysis of the green module; **(I)**, Network diagram of the top 50 genes in two groups.

To explore the interactions of these 445 ARDEGs, we conducted a PPI network using Cytoscape software. 444 nodes and 2,403 edges were obtained with *P* < 1.0 × 10^−16^. The five most significant modules identified by MCODE contain a total of 93 hub genes (Figure 2A). Meanwhile, hub genes were screened by six algorithms of the cytoHubba plugin. The first 100 genes of each algorithm were intersected with 93 genes obtained by the MCODE plugin. Finally, a total of 47 final hub genes were obtained (Figure 2B).

WGCNA is another method used for gene selection. The soft-thresholding power was 28, determined based on an R-squared cut of 0.85 (Figures 2C,D). Three main modules were identified based on average hierarchical clustering and dynamic tree clipping (Figure 2E). The green (R = 0.86, *P* = 7 × 10^−07^) and gray (R = −0.89, *P* = 6 × 10^−08^) modules exhibited the strongest correlation with LN (Figure 2F). The genes in the brown module are negatively correlated with LN (Figure 2G); The genes in the green module are positively correlated with LN (Figure 2H). The top 50 genes with the strongest correlation in each module were used for further analysis (Figure 2I).

### Further hub genes identified, and the construction of prediction models

**FIGURE 3:**
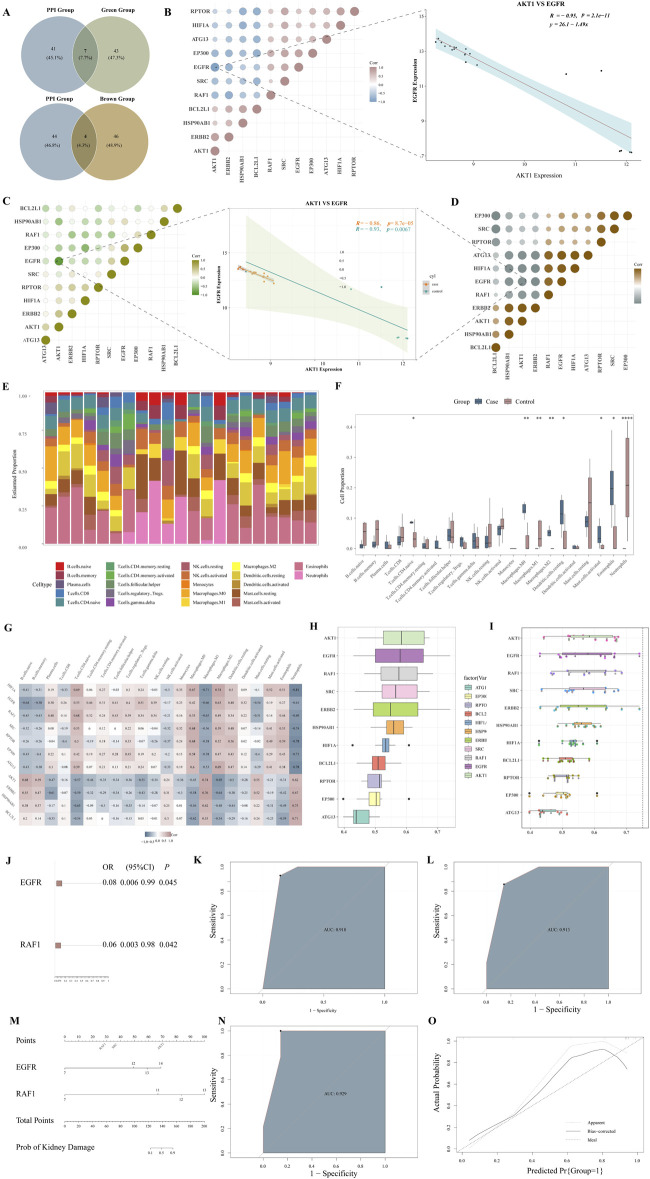
Further hub genes identified and the construction of prediction models **(A)**, Venn diagrams of PPI and green group or brown Group; **(B)**, Correlation analysis of hub genes in all samples. **(C)**, Correlation analysis of hub genes in LN samples. **(D)**, Correlation analysis of hub genes in control samples; Dot plots of correlation between expression of EGFR and AKT1 were displayed; **(E)**, Relative percentage of 22 subpopulations of immune cells in all samples; **(F)**, The box plot of the proportion of each immune cell type between healthy and LN samples; blue corresponds to cases and pink to controls, P < 0.05; **(G)**, The correlation heatmap of correlation analysis between the expression level of hub genes and the abundance of immune cell infiltration; **(H)**, Histogram of friends analysis of ARDEGs; **(I)**, Cloud and rain map of friends analysis of ARDEGs; **(J)**, Logistic regression analysis; **(K)**, the ROC analysis of EGFR; **(L)**, the ROC analysis of RAF1; **(M)**, the nomogram; **(N)**, the ROC analysis of EGFR + RAF1; **(O)**, the Calibration curve.

To refine the selection of hub genes, we integrated the results from PPI and WGCNA to identify the final set of hub genes (HIF1A, EGFR, RAF1, SRC, RPTOR, EP300, ATG13, AKT1, ERBB2, HSP90AB1, BCL2L1) (Figure 3A). The “cor” function calculates the expression correlation of the 11 ARDEGs in all control and LN samples. The expression of EGFR and AKT1 is significantly negatively correlated (all samples: R = −0.95, *P* = 2.1 × 10^−11^; control samples: R = −0.93, P = −6.7 × 10^−03^; LN samples: R = −0.86, *P* = 8.7 × 10^−05^) (Figures 3B–D).

To explore the potential immunological context of autophagy dysregulation in LN, immune infiltration analysis was performed in relation to the identified autophagy-related hub genes. The results revealed significant correlations between hub gene expression and specific immune cell populations. The CIBERSORT algorithm calculates the immune infiltration scores between the 14 LN and healthy samples. The proportion of 22 immune cells in each sample is shown in Figure 3E. The CD8 T cells, M0 macrophages, M2 macrophages, dendritic cells, and Eosinophils were more abundant in LN samples, while M1 macrophages and Neutrophils were less expressed in LN samples (Figure 3F). Further, the Spearman correlation was used to explore the relationship between hub genes and immune-infiltrated cells in LN. EGFR displayed a negative correlation with naive B cells (ρ = −0.64), M1 macrophages (ρ = −0.71), and Neutrophils (ρ = −0.81); EGFR exhibited a positive correlation with M2 macrophages (ρ = 0.74). RAF1 displayed a negative correlation with M1 macrophages (ρ = −0.65) and Neutrophils (ρ = −0.69). RAF1 positively correlated with naive CD4 T cells (ρ = 0.68) (Figure 3G).

To further investigate the significance of the hub genes, we performed a Friends analysis, which revealed that AKT1, EGFR, and RAF1 play crucial roles in regulating the expression of these genes. (Figures 3H,I).

Limited by the sample size, we first conducted a univariate analysis. The EGFR (OR: 0.08, *P* = 0.045) and RAF1 (OR: 0.06, *P* = 0.042) were statistically correlated with LN (*P* < 0.05) (Figure 3J). ROC curve was applied to evaluate each hub gene’s area under the curve (AUC) values. As we expected, these two genes displayed AUC values >0.9 (EGFR AUC:0.918; RAF1 AUC:0.913) (Figures 3K,L). For better performance in diagnosis and prediction, the nomogram was shown based on the two hub genes (Figure 3M). The nomogram presented a higher AUC value (AUC:0.929) than each hub gene, suggesting that the nomogram may have a robust diagnostic value (Figure 3N). The calibration curves showed that the predicted probability of the constructed nomogram diagnostic model was similar to the ideal model (Figure 3O).

### Pathological changes and hub gene validation

**FIGURE 4:**
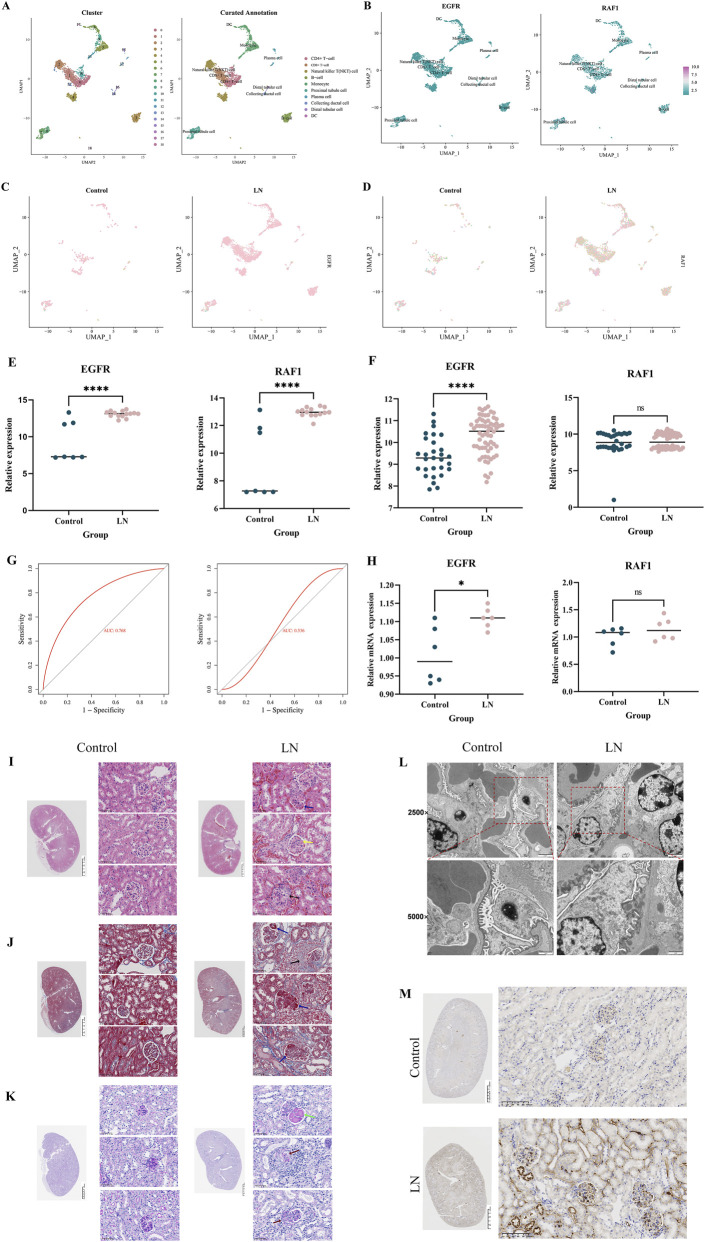
Pathological changes and hub gene validation **(A)**, The plot of cluster and curated annotation; **(B)**, The feature plot of EGFR and EGFR in LN; **(C)**, The feature plot of EGFR between control and LN; **(D)**, The feature plot of EGFR between control and LN; **(E)**, The mRNA expression of EGFR and RAF1 in the GSE112943 dataset; **(F)**, The mRNA expression of EGFR and RAF1 in the GSE32591 dataset; **(G)**, ROC analysis of EGFR (L) and RAF1 (R) in the GSE32591 dataset; **(H)**, The mRNA expression of EGFR and RAF1 by qRT-PCR (*P < 0.05,***P < 0.001, ns not significant); **(I–K)**, H&E, Masson Trichrome and PAS staining of renal tissues in the normal and lupus groups (black arrow: crescent formation within the glomerulus; blue arrow: subendothelial deposition of immune complexes; yellow arrow: hyaline thrombi in the capillary lumen; green arrow: segmental fibrinoid necrosis of the glomerulus; red arrow: fragmented cellular components within the glomerular capillary lumen); **(L)**, Podocyte alterations in the glomerulus observed by TEM; **(M)**, The expression of EGFR in control and LN by immunohistochemistry.

Combined with manual annotation of cell types, a total of 10 cell types associated with LN were identified (Figure 4A). The expression of EGFR and RAF1 was calculated. EGFR is widely expressed in various cell types. RAF1 is also expressed in multiple cell types, but the expression is lower than that of EGFR, and there is no apparent aggregation of cell types (Figure 4B). We analyzed EGFR and RAF1 in the control and LN groups to explore new biomarkers further. EGFR was obviously expressed differently in the cell types between the two groups (Figure 4C). RAF1 needs further analysis to provide a positive conclusion (Figure 4D).

Firstly, the differential expression of EGFR (*P* < 0.0001) and RAF1 (*P* < 0.0001) was demonstrated in the control and LN groups of the GSE112943 dataset. The EGFR and RAF1 were all highly expressed in the LN group (Figure 4E). Secondly, in the validation set, the differential expression of EGFR (P < 0.0001) was demonstrated in the control and LN groups, While the RAF1 was higher in LN, the statistical difference was insignificant (Figure 4F). The AUC-ROC values in the validation set were 0.768 for EGFR, 0.536 for RAF1 (Figure 4G). Further qRT-PCR analysis was conducted. Statistical analysis showed that the expression level of EGFR (P < 0.05) was higher in LN. While the RAF1 was higher in LN, the statistical difference was insignificant (Figure 4H).

Pathological results showed that, compared with the normal group, the LN group exhibited crescent formation within the glomeruli and increased deposition of immune complexes beneath the endothelial cells. There was also an increase in hyaline thrombi within the capillary lumens, segmental fibrinoid necrosis of the glomeruli, and a greater presence of fragmented cellular components within the glomerular capillary lumens (Figures 4I–K).

TEM showed that, compared with the normal group, the LN group exhibited aggravated podocyte foot process effacement and foot process disruption (Figure 4L).

Immunohistochemistry showed that EGFR was highly expressed in the LN group compared with the normal group (Figure 4M).

### Further functional analysis of the hub gene

**FIGURE 5:**
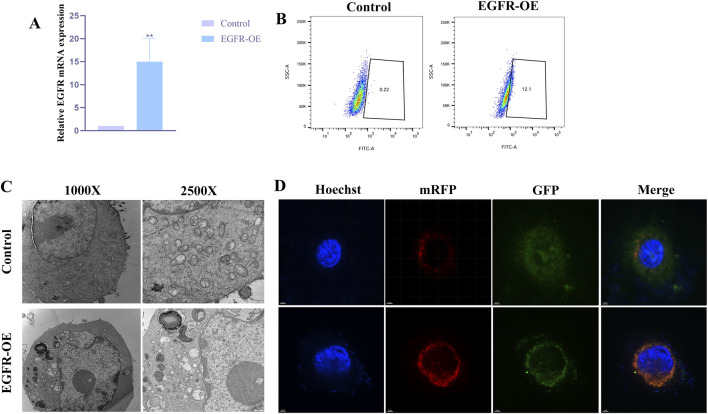
Further functional analysis of the hub gene **(A)**, Level of EGFR in CIHP-1 cells transfected with EGFR overexpressing vector or control, as determined by qRT-PCR (**P < 0.01); **(B)**, MDC fluorescence intensity with the above transfection observed by flow cytometry. **(C)**, Autophagic ultrastructures with the above transfection observed by TEM; **(D)**, Autophagic flux changes with the above transfection observed by laser confocal microscopy (yellow puncta (GFP^+^mRFP^+^) represent autophagosomes, while red puncta (GFP^-^mRFP^+^) represent autolysosomes).

To investigate the impact of aberrant EGFR activation on podocyte autophagy, we first established a CIHP-1 cell model stably overexpressing human EGFR. As shown in Figure 5A, compared with the control group infected with empty-vector lentivirus, cells infected with the EGFR-overexpression lentivirus (EGFR-OE) exhibited an approximately 20-fold increase in EGFR mRNA expression.

To evaluate the overall effect of EGFR activation on autophagy, we performed MDC staining combined with flow cytometry. Flow cytometric analysis revealed that the MDC fluorescence intensity was significantly elevated in the EGFR-OE group compared with the control group (Figure 5B). To more intuitively visualize autophagic ultrastructures, TEM was conducted for both groups. As shown in Figure 5C, typical autophagosomes were observed in the cytoplasm of EGFR-OE cells, characterized by double-membrane structures enclosing undegraded cytoplasmic components.

Laser confocal microscopy imaging showed that, compared with the control group, EGFR-OE cells exhibited a significant increase in the total number of LC3-positive puncta (yellow + red), consistent with the MDC staining and TEM results. More importantly, EGFR-OE cells displayed not only an increase in yellow puncta, but an even more pronounced increase in red puncta (Figure 5D). This finding clearly indicates that EGFR overexpression not only promotes the formation of autophagosomes but also more strongly enhances their maturation into autolysosomes.

## Discussion

With the rapid development of sequencing technologies, the molecular landscape and underlying mechanisms of various diseases have been increasingly elucidated (Wang et al., 2019; Han et al., 2021). In parallel, the reuse of publicly available sequencing datasets combined with integrated bioinformatics analyses and machine learning approaches has become an efficient strategy to identify novel genes, potential diagnostic or prognostic biomarkers, underlying mechanisms, and therapeutic targets, while reducing experimental costs (Du et al., 2022; Lian et al., 2023; Zhang, 2023). In this context, the present study applies an integrative bioinformatics framework to existing LN mRNA sequencing data, focusing specifically on autophagy-related genes to systematically characterize their potential involvement in LN pathogenesis. Importantly, compared with the previous study (Zhang et al., 2025), our work further integrates validation through cellular experiments, aiming to provide functional support for the autophagy-related genes identified by bioinformatics analyses and to deepen the understanding of the role of autophagy in LN.

Firstly, we analyzed the expression profile data of kidney tissue samples of LN and the healthy control group in the GEO database, identified the DEGs in LN, and conducted KEGG pathway enrichment analysis on the DEGs. We found that DEGs were enriched in autophagy pathways and autophagy-related pathways, such as the mTOR signaling pathway (Wang and Zhang, 2019), PI3K-Akt signaling pathway (Kma and Baruah, 2022), and MAPK signaling pathway (Zhou et al., 2015). Secondly, we obtained autophagy-related genes from autophagy-related websites and data sets and intersected with DEGs in LN to obtain ARDEGs. The obtained ARDEGs were further analyzed by GSEA and GSVA, and the functions of macroautophagy, hypoxia, and the mTOR and PI3K-Akt signaling pathways were further focused on. These results indicate that macroautophagy, hypoxia, the mTOR signaling pathway, and the PI3K-Akt signaling pathway play an essential role in LN. It has been found that under pathological conditions such as hypoxia, the relative excessive accumulation of ROS can induce autophagy.

Meanwhile, Autophagy, in turn, may reduce oxidative damage by phagocytosis and degradation of oxidizing substances (Li et al., 2015). Then, we used PPI network construction and WGCNA to obtain hub genes from ARDEGs. 11 hub genes: RPTOR, HSP90AB1, SRC, ERBB2, EP300, AKT1, RAF1, ATG13, HIF1A, EGFR, BCL2L1 were identified. Immune-infiltration analysis revealed potential associations between immune cell populations and LN pathogenesis. The balance between M1 and M2 macrophages appears to be involved in disease progression. Specifically, the hub gene EGFR showed a negative correlation with naive B cells, M1 macrophages, and neutrophils, and a positive correlation with M2 macrophages. RAF1 was negatively correlated with M1 macrophages and neutrophils, and positively correlated with naive CD4^+^ T cells. Correlation and network analyses suggest that EGFR and RAF1 may play central roles among the identified hub genes. It is important to note that these results reflect associations rather than direct causal relationships, providing insights into potential interactions between hub genes and immune cells in LN. Given the established role of autophagy in regulating immune cell survival, activation, and polarization, the observed associations between autophagy-related hub genes and immune cell infiltration may reflect potential interactions between autophagy dysregulation and the immune microenvironment in LN. Univariate analysis combined with ROC analysis shows that these two hub genes (EGFR, RAF1) displayed ideal predictive performance for LN. Moreover, two autophagy-related hub genes, EGFR and RAF1, were employed to develop a diagnostic nomogram model to predict the risk of LN. The ROC analysis and calibration curve coincide, showing the model has good predictive performance.

The official full name of EGFR is epidermal growth factor receptor. The encoded protein is a member of the protein kinase superfamily (Yarden and Sliwkowski, 2001). EGFR binds to EGF as a cell surface protein, thus inducing receptor dimerization and tyrosine autophosphorylation, leading to cell proliferation (Roepstorff et al., 2009). EGFR inhibits autophagy by up-regulating rubicon in podocytes of type 2 diabetes nephropathy (Li et al., 2021). Inhibition of EGFR can attenuate the development of diabetic nephropathy in type 1 diabetes, partly mediated by mTOR inhibition and AMPK activation, with increased autophagy (Zhang et al., 2014). EGFR mediated the renal cell apoptosis in the rhabdomyolysis-induced model via upregulation of autophagy (Sun et al., 2022). Based on previous studies on EGFR in kidney disease and our study, the relationship between EGFR and autophagy in LN can be a new research direction. Severe and pathological EGFR activation acts like a *“red alert—system overload”* crisis signal to the cell. Although growth signals are still received, the overwhelming stress signals force the cell to initiate autophagy (a “cleanup mode”) in an attempt to remove damaged components for survival. However, this type of autophagy is often dysregulated and excessive, and may ultimately lead to cell death.

Our comprehensive bioinformatics analysis, combined with experimental validation, identified key autophagy-related genes that may play central roles in the pathogenesis of LN. These findings provide new insights into the molecular mechanisms underlying LN and suggest potential biomarkers and therapeutic targets. However, several limitations should be acknowledged. The primary analysis relied on a single public dataset (GSE112943) with a limited sample size, which may affect the generalizability of our results. Although an additional dataset (GSE32591) was used for preliminary validation and the hub genes were validated at the mRNA and protein levels, further confirmation in independent cohorts or larger patient populations is still required. Additionally, although our *in vitro* experiments demonstrate that EGFR activation modulates podocyte autophagy, this study does not directly establish a causal relationship between EGFR-mediated autophagy regulation and LN progression *in vivo*. Further studies using LN animal models and pharmacological EGFR inhibition are required to validate the functional significance of EGFR–autophagy signaling in LN pathogenesis. Despite these limitations, our study offers a valuable framework for future investigations into autophagy-related mechanisms in LN and may guide the development of targeted therapeutic strategies.

The identification of EGFR as a potential key regulator of dysregulated autophagy in LN pathogenesis ties in well with existing models of aberrant apoptosis and accumulated autoantigens driving systemic autoimmunity in LN and SLE. The current finding of elevated EGFR expression disrupting autophagy homeostasis provides a compelling mechanism connecting autoantigen persistence from impaired apoptotic cell clearance to organ damage from inflammation. Targeting the autophagy-immunity axis via EGFR modulation may offer new therapeutic possibilities.

## Data Availability

The original contributions presented in the study are included in the article/supplementary material, further inquiries can be directed to the corresponding authors.
